# Chemo-radiotherapy plus durvalumab for loco-regional relapse of resected NSCLC

**DOI:** 10.1186/s13014-022-02084-5

**Published:** 2022-07-16

**Authors:** Paolo Borghetti, Jessica Imbrescia, Giulia Volpi, Vieri Scotti, Michele Aquilano, Alessio Bruni, Davide Franceschini, Stefano Ursino, Patrizia Ciammella, Gaia Piperno, Maria Taraborrelli, Stefano Maria Magrini

**Affiliations:** 1 Radiation Oncology Department, ASST Spedali Civili and Brescia University, Brescia, Italy; 2 Radiation Oncology Unit, Oncology Department AOU Careggi Firenze, Firenze, Italy; 3 Radiotherapy Unit, Department of Oncology and Hematology, University Hospital of Modena, Modena, Italy; 4 IRCCS Humanitas Research Hospital - Radiotherapy and Radiosurgery Department, Milan, Italy; 5 Radiation Oncology Unit, University Hospital Santa Chiara Pisa, Pisa, Italy; 6 Radiotherapy Unit, Department of Oncology and Advanced Technologies, AUSL-IRCCS, Reggio Emilia, Italy; 7 Division of Radiation Oncology, IEO, European Institute of Oncology IRCCS, Milan, Italy; 8 Radiation Oncology Unit, “SS Annunziata” Hospital, “G. D’Annunzio” University, Chieti, Italy

**Keywords:** Recurrence, Non-small cell lung cancer (NSCLC), Durvalumab, Chemo-radiotherapy

## Abstract

**Background:**

tumor recurrence after NSCLC surgical resection is the most common cause of treatment failure that sharply reduces the patient’s life expectancy. The optimal treatment strategy for loco-regional recurrences developing after surgical resection in patients with non–small-cell lung cancer (NSCLC) is not established yet.

This report aims to describe the pattern of relapse, PFS, and OS in patients treated with radio-chemotherapy and durvalumab for loco-regional relapse after surgery.

**Methods:**

We conducted a multicenter, retrospective study including subjects who underwent surgical resection for NSCLC and were treated with Pacific protocol after loco-regional relapse.

**Results:**

Twenty-four patients met the inclusion criteria. At the time of diagnosis mean age was 65 years (range 47–78), the majority being male (58.3%). The 12-month progression-free survival rate was 68.7%, the 18-month progression-free survival rate was 45.8%, and the 24-month progression-free survival rate was 34.3%. There were three deaths: the 12-month survival rate was 91%, and the 18-month survival rate was 82.8%.

**Conclusions:**

In this article, we propose a treatment strategy that might prolong post recurrence survival in patients with good performance status experiencing loco-regional relapse after surgery.

## Introduction

Despite progress, lung cancer is still the leading cause of cancer-related deaths [1]. Most patients are diagnosed with advanced-stage disease, as symptoms usually occur when the disease has already spread [2]. Approximately 50% of lung cancers are diagnosed at earlier clinical stages [3], and this proportion is expected to increase with the implementation of lung cancer screening programs in Europe and US. [4]. Surgical resection remains the first treatment choice for stage I-IIIA non-small cell lung cancer (NSCLC) [5, 6]. For these patients, tumor recurrence after surgical resection is the most common cause of treatment failure [7]. Post-operative failures developing after curative surgery in patients with NSCLC may be either loco-regional or systemic [8]. There is a constant frequency of loco-regional recurrence alone, and its proportion varies from 8 to 37% [9, 10]. Post-recurrence survival is largely dependent on both the mode of recurrence and the treatment modality [11]. Multimodality treatments with salvage curative intent such as sequential or concomitant chemo-radiotherapy (CRT) reported median overall survival (OS) and progression-free survival (PFS) ranging from 16 to 70 months, and from 5 to 18 months, respectively [6, 12–18].

Due to the pulmonary and hilar-mediastinal involvement, post-operative loco-regional recurrences can be considered in a way similar to unresectable stage III NSCLC [6]. Recently, the therapeutic outcomes for unresectable stage III NSCLC patients have been drastically improved due to the introduction of consolidation immunotherapy with the programmed death-ligand 1 (PD-L1) inhibitor durvalumab. This regimen, called PACIFIC-regimen, is now widely adopted as the standard-of-care [19–21]. Durvalumab is a selective, high-affinity, human immunoglobulin G1 monoclonal antibody that blocks the interaction of PD-L1 with programmed cell death protein 1 and CD80, allowing T-cells to recognize and kill tumor cells (TCs) [22]. In this retrospective study, we aim to assess this treatment strategy in a series of NSCLC patients with post-operative, loco-regional only recurrence.

## Material and methods

We conducted a multi-centre, retrospective, observational study including patients with loco-regional relapse after complete surgical resection who received CRT followed by durvalumab, between October 2018 and December 2020, in 8 Italian institutions. The ethical committee of the Coordinating Centre approved this study (NP 4634); all other Centres subsequently approved the study when requested by the local ethical committee.

Inclusion criteria: we included patients with resected NSCLC, who had a loco-regional (pulmonary and/or hilar and/or mediastinal) relapse and had received concomitant or sequential CRT followed by durvalumab for at least one administration.

The primary endpoint was median PFS. Secondary endpoints included 12, 18, and 24 months PFS rates, and median OS. PFS was defined as the time from the end of RT to the date of the first documented event of tumour progression/relapse or death. OS was defined as the time from the end of RT until death from any cause.

Patients received durvalumab from 5 to 90 days (average 29, median 25 days) after CRT completion. Durvalumab was administered intravenously at a dose of 10 mg per kilogram of body weight every 2 weeks as consolidation therapy up to 12 months. Treatment was discontinued if there was confirmed disease progression, or unacceptable toxic effects.

Data collected included age, gender, histology, type of surgery, lymphadenectomy, pathologic stage according to the TNM 8th edition, history of adjuvant chemotherapy, time to recurrence after surgery, site of recurrence, ECOG (*Eastern Cooperative Oncology Group*) performance status, radiation dose, concurrent or sequential CRT, CT regimen, and the interval between CRT and first durvalumab administration. Adverse events during CRT and after durvalumab treatment were also collected and described as defined by the Common Terminology Criteria for Adverse Events v5.0 (CTCAE) [23]

## Statistical analysis

For PFS and OS calculation we used the Kaplan–Meier method. Log-Rank test was applied to compare survival at univariate analysis, with a *p* value of < 0.05 for statistical significance.

## Result

We identified and included 24 patients, 10 females and 14 males. Median age was 65 years (range 47–78 years). Patient and disease features are summarized in Tables 1 and 2, at the time of primary surgery and at recurrence, respectively. PD-L1 expression was tested on locoregional relapses when a new biopsy was performed (12 patients, 50%). When recurrence was only radiologically proven, PD-L1 expression was evaluated on the primary surgical specimen. The case with PD-L1 negative was included in the expanded access program which also allowed patients with PD-L1 < 1% to have immunotherapy as maintenance after chemo-radiotherapy. Driver mutations were investigated in 20 patients within the 22 adenocarcinoma. KRAS mutation and ROS 1 rearrangement were found in 7 cases and 1 case, respectively. No others mutations were detected. As defined by our study’ criteria, all patients received CRT with curative intent before durvalumab.

**Table 1:** Patients’ characteristics at the time of surgery (n = 24)

	Mean	Range
Age (years)	65.29	47–78
Sex	Frequency	%
Male	14	58.3
Female	10	41.7
Histologic type	Frequency	%
Adenocarcinoma	22	91.7
Squamous cell	2	8.3
Surgery	Frequency	%
Lobectomy	20	83.5
Sublobar resection	4	16.5
Lymphadenectomy	Frequency	%
Yes	21	87.5
No	3	12.5
pT stage	Frequency	%
pT1	9	37.5
pT2	11	45.8
pT3	1	4.2
pT4	3	12.5
pN stage	Frequency	%
pN0	17	70.8
pN1	6	25.0
pN2	1	4.2
TNM Classification 8th Ed		
I	6	25.0
IIA	5	20.8
IIB	8	33.4
IIIA	5	20.8
(Neo)Adjuvant treatment at the time of surgery		
None	16	66.6
Adjuvant	4	16.7
Neoadjuvant	4	16.7

**Table 2:** Patients’ characteristics at the time of first recurrence prior to CRT plus durvalumab (n = 24)

	Mean	Range
Median disease-free interval^a^ (months)	31.29	3–185
	Frequency	%
Histological confirmation	13	54.2
Time to recurrence	Frequency	%
< 1 year	10	41.7
> 1 year	14	58.3
Pulmonary recurrence site	Frequency	%
No pulmonary site	16	66.6
At surgical staple lines	4	16.7
Other lung sites	4	16.7
Lymph nodes relapse	Frequency	%
Ipsilateral hilum	2	8.3
Ipsilateral ilo-mediastinic	18	75.0
Contralateral ilo-mediastinic	3	12.5
Supraclavicular	1	4.2
PD-L1 expression	Frequency	%
Negative	1	4.2
≤ 50%	11	45.9
> 50%	12	50.0
PD-L1 assessment	Frequency	%
At surgery	12	50.0
At recurrence	12	50.0
ECOG performance status	Frequency	%
0	15	62.5
1	8	33.3
2	1	4.2

^*a*^*From primary resection to recurrent disease diagnosis*

Table 3 reports different treatment modalities. RT average dose was 59.5 Gy, ranging between 50 and 66 Gy. The only patient treated with 50 Gy received a mild hypofractionated RT schedule (2,75 Gy per fraction), reaching an equivalent dose of 58 Gy delivered at 2 Gy per fraction. CHT regimens varied, but all contained a first platinum agent, and the most common second drug was paclitaxel followed by gemcitabine.

**Table 3:** Treatment at recurrence

CT 1° drug	Frequency	%
Cisplatin	8	33.3
Carboplatin	16	66.7
CT 2° drug	Frequency	%
Etoposide	3	12.5
Paclitaxel	9	37.5
Pemetrexed	3	12.5
Gemcitabine	5	20.8
Vinorelbine	4	16.7
CT schedule	Frequency	%
Weekly	10	41.7
Every three weeks	14	58.3
Timing of CRT	Frequency	%
Concomitant	18	75.0
Sequential	6	25.0
Total RT dose	Mean	Range
	59.5	50–66
Timing from CRT to Durvalumab (days)	Mean	Range
	29	5–90

Grade 3 or 4 adverse events occurred in only 2 patients (Table 4). The most common adverse events were esophagitis (58.3%), followed by hematological toxicity (20.8%). After durvalumab administration, six patients (25%) developed pneumonitis, but only 2 of them (8.3%) had Grade 3 (Table 5). No treatment-related deaths were recorded.

**Table 4:** Radio-chemotherapy related adverse events

	Frequency	%
Esophagitis	14	58.3
G1	7	29.2
G2	5	20.8
G3	2	8.3
Pneumonitis G1	1	4.2
Hematological toxicity	5	20.8
G1	3	12.5
G2	2	8.3

**Table 5:** Durvalumab-related adverse events

	Frequency	%
Pneumonitis	6	25.0
G1	1	4.2
G2	3	12.5
G3	2	8.3

Discontinuation of treatment occurred in 3 patients (12.5%) due to adverse events (Table 6).

**Table 6:** Causes of durvalumab discontinuation

	Frequency	%
Still ongoing	7	29.2
Adverse events	3	12.5
Disease progression	9	37.5
All dose received (as planned)	5	20.8

At the time of analysis, 10 patients (41.7%) were alive without progression, 11 patients (45.8%) alive with disease recurrence, and 3 patients (12.5%) were dead (all non cancer-related deaths).

**Fig. 1:**
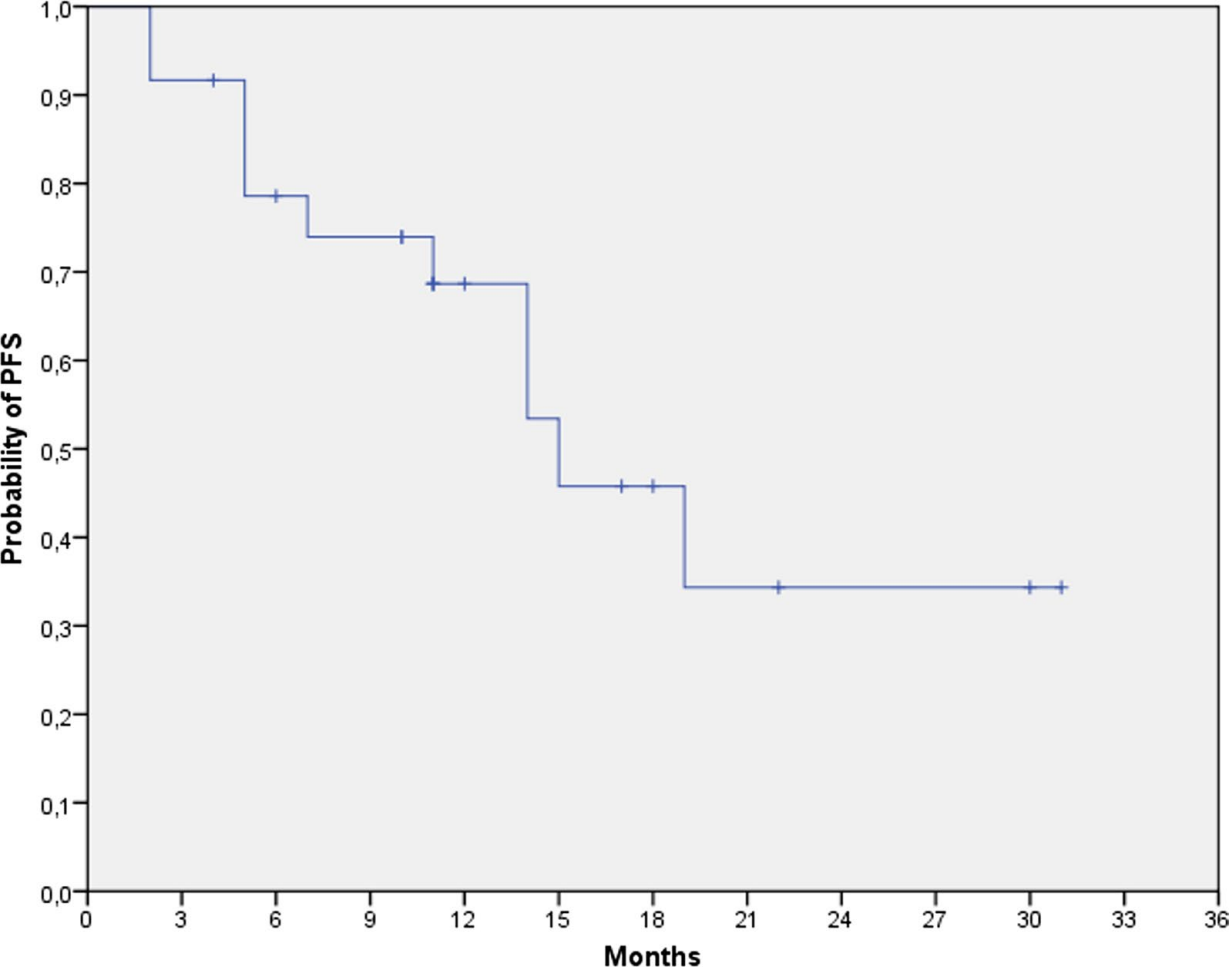
Progression-free survival in treated population. Kaplan–Meier curve for progression-free survival (PFS). Tick marks indicate censored observations, and vertical lines indicate the times of landmark PFS analyses

We recorded 14 progressions (7 local and 7 distant). The median progression-free survival was 15 months; the 12-, 18- and 24- months progression-free survival rate was 68.7, 45.8 and 34.3% (Fig. 1).

When splitting the population into two different groups according to the time to recurrence (< 12 months vs. > 12 months from surgery), the two cohorts showed similar outcomes in terms of PFS at univariate analysis. Patients with recurrence within or beyond 12 months from surgery had median PFS of 14 months and 15 months (*p* = 0.119).

## Discussion

In this brief report, we described survival outcomes and toxicity following the application of PACIFIC-like regimens at first recurrence after surgery in a series of 24 patients. To the best of our knowledge, this is the first report describing the safety and results of CRT plus durvalumab in this setting. All patients referred to this combination outside a clinical trial were extremely selected, and all have been discussed by a multi-disciplinary tumor board.

Radiation therapy, alone or in combination with chemotherapy, is considered the standard therapeutic choice for these patients. RT alone has been reported to yield good local response rates and significant prolongation of survival [24, 25].Kelsey et al. reviewed the efficacy of RT for recurrent NSCLC, reporting a median survival time between 11 and 19 months [26, 27].

When adding chemotherapy, in the retrospective study performed by Nakamichi [22], the median PFS rate in the CRT group was 19.0 months. In a similar study by Takenada et al. [28] concurrent CRT produced a median disease-free survival of 13 months and a median post recurrence survival of 31 months.

The use of concurrent CRT might increase the local control over RT alone as well as reduce the rate of distant metastasis, as shown by Nakamichi et al. [29, 30]. Recently, Terada et al. [31] Investigated RT efficacy and safety for patients with local recurrence after lobectomy with hilar and mediastinal lymph node dissection for NSCLC, showing that the most common pattern of failure is distant metastasis, regardless of the initial recurrence site. This is the main rationale for potentiating systemic therapy in this setting and adding maintenance immunotherapy.

Patients with loco-regional relapse after surgery were not enrolled in the PACIFIC trial, although their characteristics may be considered similar to those of unresectable stage III patients.

Concurrent CRT is the standard treatment strategy employed in patients affected by unresectable locally advanced stage III NSCLC, but also a sequential approach of CHT and RT was frequently used [32].

Acknowledging the limitations of our study, mainly its retrospective nature, with uncontrolled selection biases and the small sample size, we observed promising PFS and OS results in this setting.

The data reported on the PD-L1 expression represents the only available value. In 12 patients (50%), PD-L1 expression was tested on the most recent biopsy when locoregional relapses were histologically proven. PD-L1 expression was evaluated on the surgical specimen for patients treated with radiological diagnosis of recurrence. Due to the long interval between primary surgery and locoregional recurrence, PD-L1 had never been tested prior to relapse for most patients. The prognostic and predictive impact of the PD-L1 changes over time is a critical and extremely current issue that need to be investigated in dedicated trials. Unfortunately, an extimation of the different impact of PD-L1 expression assessed in different moment cannot be performed for missing or unbtainable data. Patients included in this series were treated on the basis of the PD-L1 value available, therefore this point is an unmet need that this report cannot solve.

Due to the small sample we did not speculate on possible correlation between PFS and the clinical and therapeutic reported variables, with the exception of time to recurrence from surgery using 12 months as cut-off according to literature data and clinical sense [24].

Surprisingly, in this series 92% of cases were adenocarcinoma and KRAS mutation and ROS1 rearrangement was reported in 7 cases and 1 case, respectively. No other driver mutations were found, maybe due to the alternative therapeutic proposal offered to the patients relapsed after surgery with targetable mutations. KRAS-mutant NSCLC is associated with smoking. The efficacy of chemotherapy in patients with KRAS-mutant NSCLC is generally poor and numerous novel therapeutic strategies have been developed. Of these, immunotherapy may be one of the most promising treatment approaches for patients with KRAS-mutant NSCLC [33]. This result should be interpreted with great caution given the small sample size. However, it could be interesting to investigate the post-surgical failure pattern of mutated KRAS adenocarcinoma also in order to personalize adjuvant locoregional treatments.In the PACIFIC study, the median PFS for the experimental arm was slightly higher than how reported in this one (16.8 vs. 15 months), mainly due to the number of local and distant failures which is higher than those reported in the pivotal study (58.3% vs. 22.5%) [19, 20]. However, the latter data would be interpreted taking into consideration the setting of patients with recurrence after surgery included in this study and the relatively short follow up. The data obtained on the safety of the treatment performed are comforting, in particular the all grade and G3 pneumonitis are lower than the PACIFIC trial.

After the revolutionary paradigm shift with the advent of Durvalumab in the treatment of patients with unresectable stage III NSCLC, several real life series have been published showing how the use of immunotherapy also in this setting has invaded clinical practice [34]. This experience has shown how selected cases of patients with local recurrence after surgery could be considered in the same way as patients who are candidates for radio-chemotherapy and immunotherapy in the pacific trial.

## Conclusions

CRT followed by consolidative durvalumab for loco-regional recurrence after primary surgery seems to obtain promising results in terms of PFS, similar to those obtained in "de novo" stage III unresectable NSCLC. Indeed, this cohort is characterized by a relatively slow disease course, and it was carefully selected. Therefore, the results of this preliminary analysis should not generalized and might be not reproducible; however, for selected patients the addition of durvalumab to CRT may prolong survival over historical data and deserve further testing in prospective trials.

## Data Availability

The datasets used and/or analysed during the current study are available from the corresponding author on reasonable request.
